# Impact of greenhouse gases and air pollutants on the incidence and mortality of HBV and HCV in China

**DOI:** 10.3389/fpubh.2026.1778170

**Published:** 2026-04-10

**Authors:** Shengfei Pei, Fan Shi, Qinglin Li, Bo Zhu, Shumei Huang, Xin Zhang

**Affiliations:** 1 Changqing University Science Park, Shandong Women's University, Jinan, China; 2 School of Basic Medical Sciences, Health Science Center, North China University of Science and Technology, Tangshan, China; 3 Department of Public Health, Tianjin Union Medical Center (First Affiliated Hospital of Nankai University), Tianjin, China; 4 The First Affiliated Hospital of Shandong First Medical University, Jinan, China; 5 Hebei Key Laboratory of Immune Mechanism of Major Infectious Diseases and New Technology of Diagnosis and Treatment, The Fifth Hospital of Shijiazhuang, Shijiazhuang, China; 6 Department of Tuberculosis, The Fifth Hospital of Shijiazhuang, Shijiazhuang, China

**Keywords:** Bayesian kernel machine regression, gradient boosted regression trees, greenhouse gases, mixtures analysis, viral hepatitis

## Abstract

**Background:**

Previous studies have found that meteorological conditions are associated with an increased risk of viral hepatitis. However, other environmental factors, such as greenhouse gases and air pollutants, that may influence this risk have not yet been identified.

**Materials and methods:**

The epidemiological characteristics of major viral hepatitis, including hepatitis B virus (HBV) and hepatitis C virus (HCV), in China were investigated. Quantile-based g-computation (Qgcomp) and Bayesian kernel machine regression (BKMR) were then applied sequentially to explore the single and mixed effects of greenhouse gases and air pollutants, as well as their susceptibility associations with HBV and HCV morbidity and mortality across different age groups. Finally, the gradient boosting regression tree (GBRT) model was used to evaluate the prediction performance for different types of hepatitis.

**Results:**

Regarding the mixed effects, environmental factors that showed a positive dose–response relationship with HBV morbidity and mortality were mainly concentrated in patients aged 35-64 years and older. For HCV subtypes, there was a positive dose–response relationship between environmental factors and morbidity and mortality, except for patients aged 0-14 years. When considering single effects, NH_3_ (P25-P50) at a low concentration level showed a significant positive association with HBV pathogenesis, while CO (P5-P25) at a low concentration level and non-methane volatile organic compound (NMVOC) at all levels showed a significant negative association with HBV pathogenesis. CO_2_bio (P25-P95) showed significant positive associations with HBV mortality at nearly all concentration levels. The associations with HBV mortality decreased with increasing concentration (maximum association (95%CI) =11.54 (4.49, 18.60)), while NMVOC and CH_4_ showed significant negative associations with HBV mortality at higher concentration levels (P25-P75). CO_2_ and NMVOC at all concentration levels and NH_3_ at a moderate level showed significant positive associations with HCV incidence, while CO at all concentration levels showed significant negative associations with HCV incidence. The results based on environmental factors showed that the best predictive performance was observed for HCV (R^2^Training=0.950, R^2^Test=0.942), followed by HBV mortality.

**Conclusion:**

We found that most greenhouse gases and air pollutants were associated with increased morbidity and mortality of HBV and HCV in China. These findings may have important implications for the development of effective public health interventions and integrated early warning systems for air pollution and viral hepatitis.

## 1 Introduction

Viral hepatitis is a major global public health problem, affecting hundreds of millions of people and causing high levels of morbidity and mortality worldwide. The five liver-specific viruses—hepatitis A virus (HAV), hepatitis B virus (HBV), hepatitis C virus (HCV), hepatitis D virus (HDV), and hepatitis E virus (HEV)—each exhibit distinct epidemiology, modes of transmission, endemic patterns, and responses to antiviral therapy (1). HBV and HCV can lead to acute/chronic hepatitis, cirrhosis, and eventually liver cancer (2, 3). Approximately 60% of liver cancers occur in patients with HBV and/or HCV infection (4). In a global burden of disease assessment measured by disability-adjusted life years (DALYs), HBV and HCV accounted for the largest proportion of DALYs within the chronic liver disease category (5). According to Global Hepatitis 2024, hepatitis B and C cause approximately 3,500 deaths each day, and the mortality rate is rising. It is estimated that 254 million people worldwide have hepatitis B, 50 million have hepatitis C, and 6,000 people are newly infected with viral hepatitis daily (6). China, the world’s most populous developing country and the second-largest economy, has attracted immigrants from many countries and regions, some of whom may carry a higher burden of hepatitis. Given that a significant portion of the global disease burden from hepatitis viruses is concentrated in China (7), addressing hepatitis virus infections in China is critical to global control strategies (8).

China, the focus of this study, is a country largely characterized by physical (industrial) manufacturing. Although the country has advocated environmental protection measures in recent years, some environmental pollution still persists. Environmental change is a major risk factor affecting human health. Rising environmental temperatures are often primarily caused by increased greenhouse gases that trap excess heat in the atmosphere, leading to conditions such as erratic weather patterns, wildfires, and the spread of vector-borne diseases, and these greenhouse gases set the stage for new devastating health threats across the globe (9). A study in Taiwan showed that long-term exposure to PM_2.5_ increases the risk of liver cancer, and chronic inflammation of the liver may underlie its pathogenesis (10). However, the existing evidence lacks exploration of the different types of viral hepatitis and the combined effects of greenhouse gases and air pollution on viral hepatitis morbidity and mortality.

Due to the informatization requirements of the medical system, medical records and data are widely available in hospitals and primary care institutions. However, due to the preprocessing requirements of the original data, the data utilization rate is still very low, and this poor data quality limits the data utilization rate (11). A recent study in Hong Kong, China, showed that ridge regression and random forest models are effective in predicting the onset of hepatocellular carcinoma in patients with chronic viral hepatitis (12). With their unique algorithmic approaches, machine learning (ML) techniques are increasingly used to extract valuable information from medical data and build advanced models to assist clinical practice (13).

Our specific objectives were to: (a) to explore the epidemic characteristics of viral hepatitis in China, (b) assess the effects of greenhouse gases and air pollutants on HBV and HCV morbidity and mortality, and (c) establish and validate HBV and HCV case reporting and mortality surveillance models.

## 2 Materials and methods

### 2.1 Data source

All recorded cases of viral hepatitis in China from 2005 to 2018 were obtained from the Public Health Sciences Data Center website (https://www.phsciencedata.cn/). The website provides data on the incidence and morbidity of patients with different types of viral hepatitis. All patients were diagnosed according to the criteria of viral hepatitis management issued by the Ministry of Health of the People’s Republic of China. Greenhouse gas and air pollutant data (2005–2018) were obtained from the Emissions Database for Global Atmospheric Research (EDGAR) (https://edgar.jrc.ec.europa.eu/). Greenhouse gases included CH_4_, CO_2_bio, N_2_O, and CO_2_. Air pollutants included black carbon (BC), CO, NH_3_, non-methane volatile organic compound (NMVOC), Nox, organic carbon (OC), PM_2.5_, PM_10_, and SO_2_.

### 2.2 Pollution mixture analysis across different statistical models

In this study, multiple mixture models were used to investigate the overall effects, weights, and individual effects of greenhouse gases and air pollutants on the morbidity and mortality of the major subtypes of viral hepatitis (HBV and HCV), as well as to compare their effects. All model analyses were performed using R (version 4.4.1, R Core Team, Vienna, Austria). The application model combines the inferential simplicity of weighted quantiles and regression with the flexibility of quantile-based g-computation. Qgcomp is a simple and computationally efficient method for estimating associations between exposure combinations and expected health outcomes (14). To fit a linear model of cognitive function, we used the qgcomp.noboot function to assess the total effect by partitioning each greenhouse gas and air pollutant into quartiles and assigning a positive or negative index weight to each exposure. We then used the qgcomp.boot function to determine the linearity of the mixture effect, which was visualized using g-computation (R package, “qgcomp”).

Due to the strong correlations between some gases and pollutants, Bayesian kernel machine regression (BKMR) models were used to assess their individual and cumulative effects on morbidity and mortality in major viral hepatitis subtypes (HBV and HCV). The basic principle of the method is to estimate the multiple mixed exposure–response relationship (R package, “bkmr”) by introducing a kernel function based on the covariance (inner product) among individual greenhouse gas and pollutant exposures (15). In the BKMR model, covariates and main effects were referenced from the positive and negative effects obtained using the Qgcomp model. Due to significant differences in units and magnitudes between greenhouse gases and air pollutants, all exposure variables were standardized using the scale function prior to BKMR modeling.

### 2.3 Prediction training process

To predict different types of viral hepatitis, we applied the gradient boosting regression tree (GBRT) algorithm and compared the results. To ensure consistency, each model was trained on 75% of the training sample. A common 25% holdout set was retained for all models and used to generate statistics for comparing results between them. The GBRT model primarily relies on generalized step-up regression modeling and uses iterative methods to increase the deviation function, so as to establish the optimization objective (R package, “gbm”) according to the given loss function. The prediction function was selected using 5-fold cross-validation. All algorithm analyses in this study were performed using R (version 4.4.1, R Core Team, Vienna, Austria).

## 3 Results

### 3.1 Epidemiological characteristics of hepatitis viruses, greenhouse gases, and pollutants nationwide

**Figure 1:**
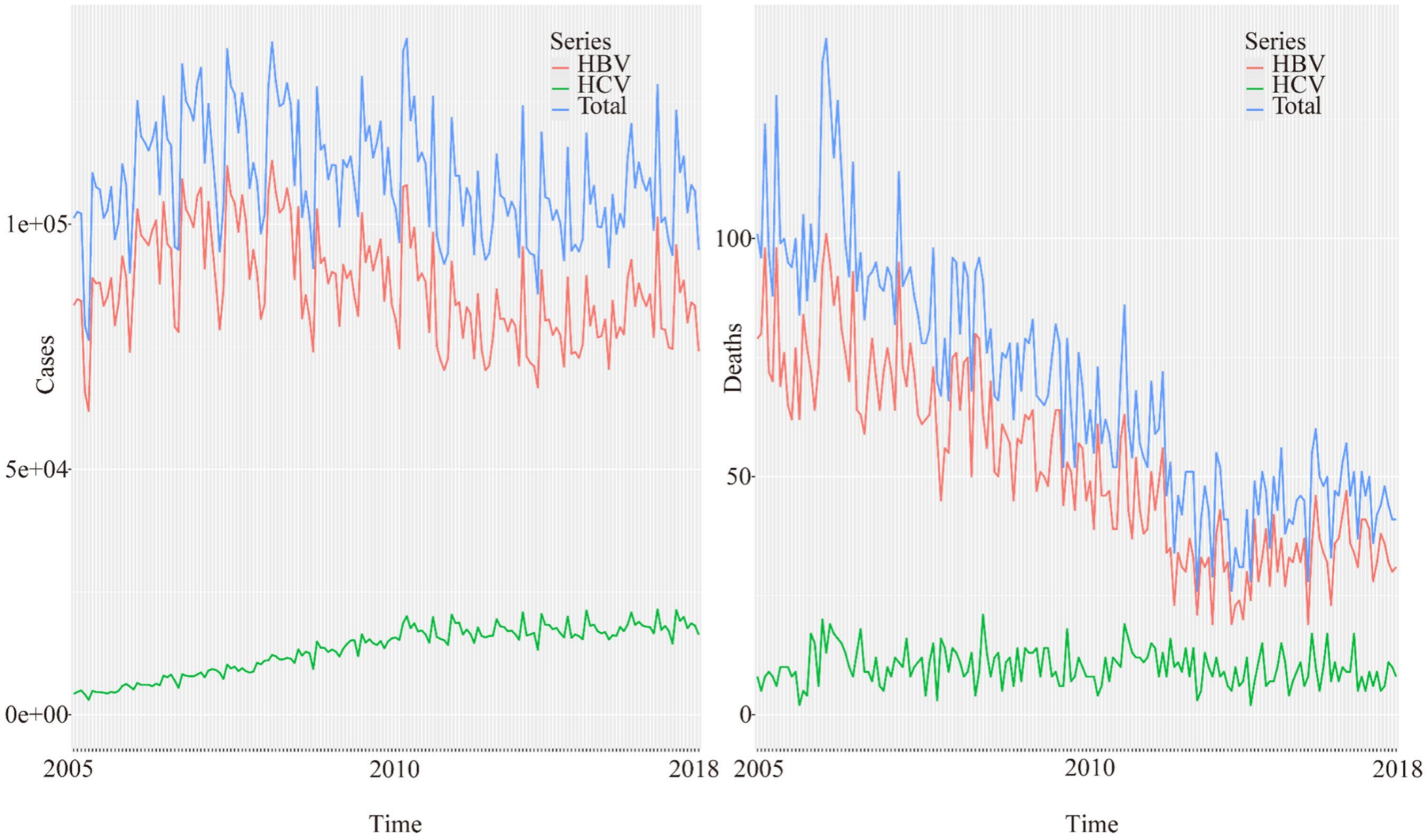
Epidemiological trends and characteristics of reported incidence and mortality of viral hepatitis and its major types (HBV and HCV) in China from 2005 to 2018.

As shown in Figure 1, the overall reported cases of viral hepatitis in China from 2005 to 2018 showed a steady epidemic trend, first increasing and then decreasing, while the number of deaths experienced a sharp short-term rise followed by a significant decline. The trend line also displayed seasonal variations. Among the subtypes, HBV accounted for the majority of cases and deaths, showing similar trends. Overall, the incidence and mortality of HCV have been rising. However, the number of reported HCV cases and deaths remains much lower than that of HBV.

According to the incidence and mortality data for HBV presented in Table 1, during the period 2005–2018, the prevalence rate of HBV decreased from 6.30/10^5^ to 6.00/10^5^ and the mortality rate decreased from 0.0058/10^5^ to 0.0024/10^5^. Among the cases, the most affected age group was 35–64 years, accounting for 50.73%, followed by those aged 15–34 years, who accounted for 38.32%. Regarding mortality, the most affected age group was 35–64 years, accounting for 67.20%, followed by those over 65 years, who accounted for 19.85%. Regarding seasonal patterns, the incidence and mortality of HBV were highest in spring and summer, accounting for 52.62 and 50.40%, respectively. According to the incidence and mortality data for HCV presented in Table 2, during the epidemic period 2005–2018, the prevalence rate of HCV increased from 0.34/10^5^ to 1.32/10^5^ and the mortality rate decreased from 0.0006/10^5^ to 0.0005/10^5^. Among the cases, the most affected age group was 35–64 years, accounting for 61.71%, followed by those over 65 years, who accounted for 19.53%. Regarding mortality, the most affected age group was 35–64 years, accounting for 56.90%, followed by those over 65 years old, who accounted for 30.31%. Regarding seasonal characteristics, HCV cases were primarily concentrated in spring and summer, while deaths were primarily concentrated in winter.

**Table 1:** Distribution of HBV cases and deaths by age group and season in China, 2005–2018.

Series	Characteristic		0–14	15–34	35–64	65+	Total	Rate (/10^5^)
			No. of HBV cases (%)					
HBV cases	Year	2005	54,593 (5.56%)	481,435 (49.01%)	398,266 (40.54%)	48,004 (4.89%)	982,298	6.30
		2006	52,161 (4.70%)	532,767 (48.03%)	466,512 (42.06%)	57,690 (5.21%)	1,109,130	7.07
		2007	44,497 (3.81%)	535,949 (45.81%)	521,736 (44.59%)	67,764 (5.79%)	1,169,946	7.42
		2008	35,328 (3.03%)	510,924 (43.68%)	548,893 (46.93%)	74,424 (6.36%)	1,169,569	7.38
		2009	30,467 (2.58%)	490,342 (41.57%)	574,905 (48.74%)	83,893 (7.11%)	1,179,607	7.40
		2010	20,395 (1.92%)	415,726 (39.20%)	540,226 (50.94%)	84,235 (7.94%)	1,060,582	6.62
		2011	15,844 (1.45%)	420,462 (38.45%)	565,230 (51.70%)	91,799 (8.40%)	1,093,335	6.79
		2012	24,609 (2.27%)	416,615 (38.32%)	551,483 (50.73%)	94,379 (8.68%)	1,087,086	6.72
		2013	10,622 (1.10%)	338,672 (35.17%)	520,114 (54.01%)	93,566 (9.72%)	962,974	5.93
		2014	9,379 (1.00%)	315,302 (33.70%)	513,804 (54.91%)	97,217 (10.39%)	935,702	5.75
		2015	8,894 (0.96%)	296,522 (31.74%)	523,853 (56.07%)	104,946 (11.23%)	934,215	5.71
		2016	7,886 (0.84%)	289,630 (30.74%)	533,918 (56.66%)	110,834 (11.76%)	942,268	5.73
		2017	8,313 (0.83%)	290,631 (29.01%)	576,726 (57.56%)	126,282 (12.60%)	1,001,952	6.05
		2018	8,150 (0.82%)	271,319 (27.13%)	585,528 (58.55%)	134,988 (13.50%)	999,985	6.00
	Seasons	Spring (March–May)	83,561 (2.12%)	1,489,776 (37.74%)	2,023,369 (51.25%)	351,090 (8.89%)	3,947,796	
		Summer (June–August)	96,805 (2.59%)	1,474,522 (39.32%)	1,865,023 (49.73%)	313,614 (8.36%)	3,749,964	
		Autumn (September–November)	76,917 (2.26%)	1,306,952 (38.23%)	1,733,979 (50.72%)	300,549 (8.79%)	3,418,397	
		Winter (December–February)	73,855 (2.10%)	1,335,046 (38.01%)	1,798,823 (51.21%)	304,768 (8.68%)	3,512,492	
	Total		331,138 (2.27%)	5,606,296 (38.32%)	7,421,194 (50.73%)	1,270,021 (8.68%)	14,628,649	
HBV deaths	Year	2005	6 (0.66%)	141 (15.53%)	598 (65.86%)	163 (17.95%)	908	0.0058
		2006	12 (1.21%)	169 (16.98%)	675 (67.84%)	139 (13.97%)	995	0.0063
		2007	4 (0.47%)	121 (14.17%)	573 (67.10%)	156 (18.26%)	854	0.0054
		2008	4 (0.48%)	112 (13.48%)	560 (67.39%)	155 (18.65%)	831	0.0052
		2009	7 (0.89%)	89 (11.24%)	534 (67.42%)	162 (20.45%)	792	0.0050
		2010	5 (0.73%)	86 (12.48%)	479 (69.52%)	119 (17.27%)	689	0.0043
		2011	2 (0.32%)	85 (13.34%)	434 (68.13%)	116 (18.21%)	637	0.0040
		2012	2 (0.35%)	74 (12.71%)	390 (67.01%)	116 (19.93%)	582	0.0036
		2013	2 (0.36%)	51 (9.27%)	399 (72.55%)	98 (17.82%)	550	0.0034
		2014	0 (0.00%)	35 (9.72%)	243 (67.50%)	82 (22.78%)	360	0.0022
		2015	2 (0.57%)	32 (9.09%)	235 (66.76%)	83 (23.58%)	352	0.0022
		2016	0 (0.00%)	37 (9.14%)	262 (64.69%)	106 (26.17%)	405	0.0025
		2017	1 (0.24%)	33 (7.76%)	262 (61.65%)	129 (30.35%)	425	0.0026
		2018	1 (0.25%)	25 (6.05%)	265 (64.16%)	122 (29.54%)	413	0.0024
	Seasons	Spring (March–May)	15 (0.68%)	274 (12.26%)	1,501 (67.19%)	444 (19.87%)	2,234	
		Summer (June–August)	13 (0.59%)	275 (12.51%)	1,465 (66.65%)	445 (20.25%)	2,198	
		Autumn (September–November)	11 (0.50%)	265 (12.09%)	1,502 (68.52%)	414 (18.89%)	2,192	
		Winter (December–February)	9 (0.42%)	276 (12.72%)	1,441 (66.44%)	443 (20.42%)	2,169	
	Total		48 (0.55%)	1,090 (12.40%)	5,909 (67.20%)	1746 (19.85%)	8,793	

**Table 2:** Distribution of HCV cases and deaths by age group and season in China, 2005–2018.

Series	Characteristic		0–14	15–34	35–64	65+	Total	Rate (/10^5^)
			No. of HCV cases (%)					
HCV cases	Year	2005	1,185 (2.24%)	14,291 (27.00%)	27,225 (51.44%)	10,226 (19.32%)	52,927	0.34
		2006	1748 (2.47%)	18,249 (25.82%)	37,196 (52.63%)	13,488 (19.08%)	70,681	0.45
		2007	2,113 (2.29%)	23,087 (24.99%)	50,041 (54.17%)	17,137 (18.55%)	92,378	0.59
		2008	2,145 (1.98%)	25,543 (23.55%)	60,967 (56.22%)	19,791 (18.25%)	108,446	0.68
		2009	2047 (1.55%)	28,309 (21.47%)	76,203 (57.80%)	25,290 (19.18%)	131,849	0.83
		2010	2,309 (1.51%)	31,622 (20.66%)	90,036 (58.83%)	29,072 (19.00%)	153,039	0.96
		2011	2,435 (1.40%)	34,413 (19.79%)	105,853 (60.88%)	31,171 (17.93%)	173,872	1.08
		2012	2,546 (1.27%)	35,269 (17.49%)	124,424 (61.71%)	39,383 (19.53%)	201,622	1.25
		2013	2,519 (1.24%)	35,840 (17.64%)	128,311 (63.16%)	36,485 (17.96%)	203,155	1.25
		2014	2,213 (1.09%)	33,246 (16.39%)	130,540 (64.37%)	36,804 (18.15%)	202,803	1.25
		2015	2085 (1.01%)	30,877 (14.85%)	134,972 (64.92%)	39,963 (19.22%)	207,897	1.27
		2016	1888 (0.92%)	29,390 (14.21%)	133,910 (64.74%)	41,644 (20.13%)	206,832	1.26
		2017	1,634 (0.77%)	27,274 (12.74%)	138,472 (64.70%)	46,643 (21.79%)	214,023	1.29
		2018	1,381 (0.63%)	24,215 (11.04%)	143,536 (65.43%)	50,243 (22.90%)	219,375	1.32
	Seasons	Spring (March–May)	7,145 (1.17%)	104,100 (16.99%)	378,771 (61.81%)	122,713 (20.03%)	612,729	
		Summer (June–August)	7,624 (1.36%)	101,382 (18.04%)	346,832 (61.70%)	106,271 (18.90%)	562,109	
		Autumn (September–November)	6,497 (1.22%)	93,192 (17.57%)	326,768 (61.61%)	103,955 (19.60%)	530,412	
		Winter (December–February)	6,982 (1.31%)	92,951 (17.42%)	329,315 (61.71%)	104,401 (19.56%)	533,649	
	Total		28,248 (1.27%)	391,625 (17.49%)	1,381,686 (61.71%)	437,340 (19.53%)	2,238,899	
HCV deaths	Year	2005	3 (3.23%)	20 (21.51%)	35 (37.63%)	35 (37.63%)	93	0.0006
		2006	3 (1.88%)	29 (18.12%)	84 (52.50%)	44 (27.50%)	160	0.0010
		2007	3 (2.61%)	17 (14.78%)	62 (53.91%)	33 (28.70%)	115	0.0007
		2008	3 (2.44%)	24 (19.51%)	56 (45.53%)	40 (32.52%)	123	0.0008
		2009	2 (1.42%)	21 (14.89%)	78 (55.32%)	40 (28.37%)	141	0.0009
		2010	0 (0.00%)	13 (10.16%)	73 (57.03%)	42 (32.81%)	128	0.0008
		2011	0 (0.00%)	14 (11.20%)	72 (57.60%)	39 (31.20%)	125	0.0008
		2012	2 (1.85%)	11 (10.19%)	63 (58.33%)	32 (29.63%)	108	0.0006
		2013	2 (1.31%)	7 (4.58%)	95 (62.08%)	49 (32.03%)	153	0.0009
		2014	4 (3.31%)	15 (12.40%)	78 (64.46%)	24 (19.83%)	121	0.0007
		2015	1 (1.05%)	7 (7.37%)	58 (61.05%)	29 (30.53%)	95	0.0006
		2016	1 (0.93%)	6 (5.56%)	68 (62.95%)	33 (30.56%)	108	0.0007
		2017	0 (0.00%)	3 (2.50%)	85 (70.83%)	32 (26.67%)	120	0.0007
		2018	1 (1.01%)	4 (4.04%)	54 (54.55%)	40 (40.40%)	99	0.0005
	Seasons	Spring (March–May)	10 (2.43%)	37 (8.98%)	235 (57.04%)	130 (31.55%)	412	
		Summer (June–August)	8 (1.94%)	56 (13.59%)	221 (53.64%)	127 (30.83%)	412	
		Autumn (September–November)	3 (0.74%)	51 (12.53%)	244 (59.95%)	109 (26.78%)	407	
		Winter (December–February)	4 (0.87%)	47 (10.26%)	261 (56.99%)	146 (31.88%)	458	
	Total		25 (1.48%)	191 (11.31%)	961 (56.90%)	512 (30.31%)	1,689	

According to the results of Spearman correlation analysis shown in Figure S1, the following factors were identified: Among those related to HBV cases, the greenhouse gases included N_2_O and CO_2_, while the air pollutants included CO, NH_3_, and NMVOC. Among the factors associated with HBV-related mortality and HCV cases, the greenhouse gases included CH_4_, CO_2_bio, N_2_O, and CO_2_, while the air pollutants included CO, NH_3_, NMVOC, and NOx. For HCV-related mortality, the relevant greenhouse gases were CO_2_bio and N_2_O, and the air pollutants included BC, NH_3_, NOx, OC, PM_2.5_, PM_10,_ and SO_2_. In the interactions among environmental variables, all pairs showed positive correlations except for CH_4_ and certain air pollutants, which showed negative correlations (r∈ (−0.17, −0.81), *p* < 0.01).

### 3.2 Combined effects of multiple greenhouse gases and pollutants on hepatitis viruses

#### 3.2.1 Combined effects of multiple greenhouse gases and pollutants on hepatitis viruses: Qgcomp model

According to Table 3, except for patients over 65 years, the mixture of greenhouse gases and air pollutants had a significant positive effect on the incidence of HBV (log (RR) (bootstrap CI): 673.5(373.9, 973.1)), whereas all other effects were negative. For HCV morbidity, the combination showed a significant effect. In contrast, for HCV mortality, the combination showed significant effects only in the overall population and in the 35–64 age group.

**Table 3:** Comparison of mixture analysis results for HBV and HCV across different age groups.

Diseases	Group	Mixture log (RR) (bootstrap CI)	*p*
HBV cases	Total	**−2510.3 (−4957.3, −63.4)**	**0.046**
	Age 0–14	**−880.9 (−1095.8, −665.9)**	**0.000**
	Age 15–34	**−3612.5 (−4787.0, −2437.9)**	**0.000**
	Age 35–64	1309.6 (−61.0, 2680.2)	0.063
	Age 65+	**673.5 (373.9, 973.1)**	**0.000**
HBV deaths	Total	**−12.9 (−17.7, −8.1)**	**0.000**
	Age 0–14	−0.01 (−0.30, 0.28)	0.968
	Age 15–34	**−3.2 (−4.4, −2.0)**	**0.000**
	Age 35–64	**−7.3 (−11.0, −3.6)**	**0.000**
	Age 65+	**−2.4 (−3.6, −1.2)**	**0.000**
HCV cases	Total	**4430.5 (3341.4, 5519.6)**	**0.000**
	Age 0–14	**21.0 (5.5, 36.5)**	**0.009**
	Age 15–34	**547.9 (386.0, 709.7)**	**0.000**
	Age 35–64	**3099.9 (2379.2, 3820.5)**	**0.000**
	Age 65+	**761.8 (523.1, 1000.4)**	**0.000**
HCV deaths	Total	**1.5 (0.8, 2.2)**	**0.000**
	Age 0–14	−0.02 (−0.09, 0.04)	0.510
	Age 15–34	0.1 (−0.1, 0.4)	0.309
	Age 35–64	**1.1 (0.6, 1.6)**	**0.000**
	Age 65+	0.3 (−0.1, 0.6)	0.093

Bold font indicates statistical significance at the 0.05 level.

**Figure 2:**
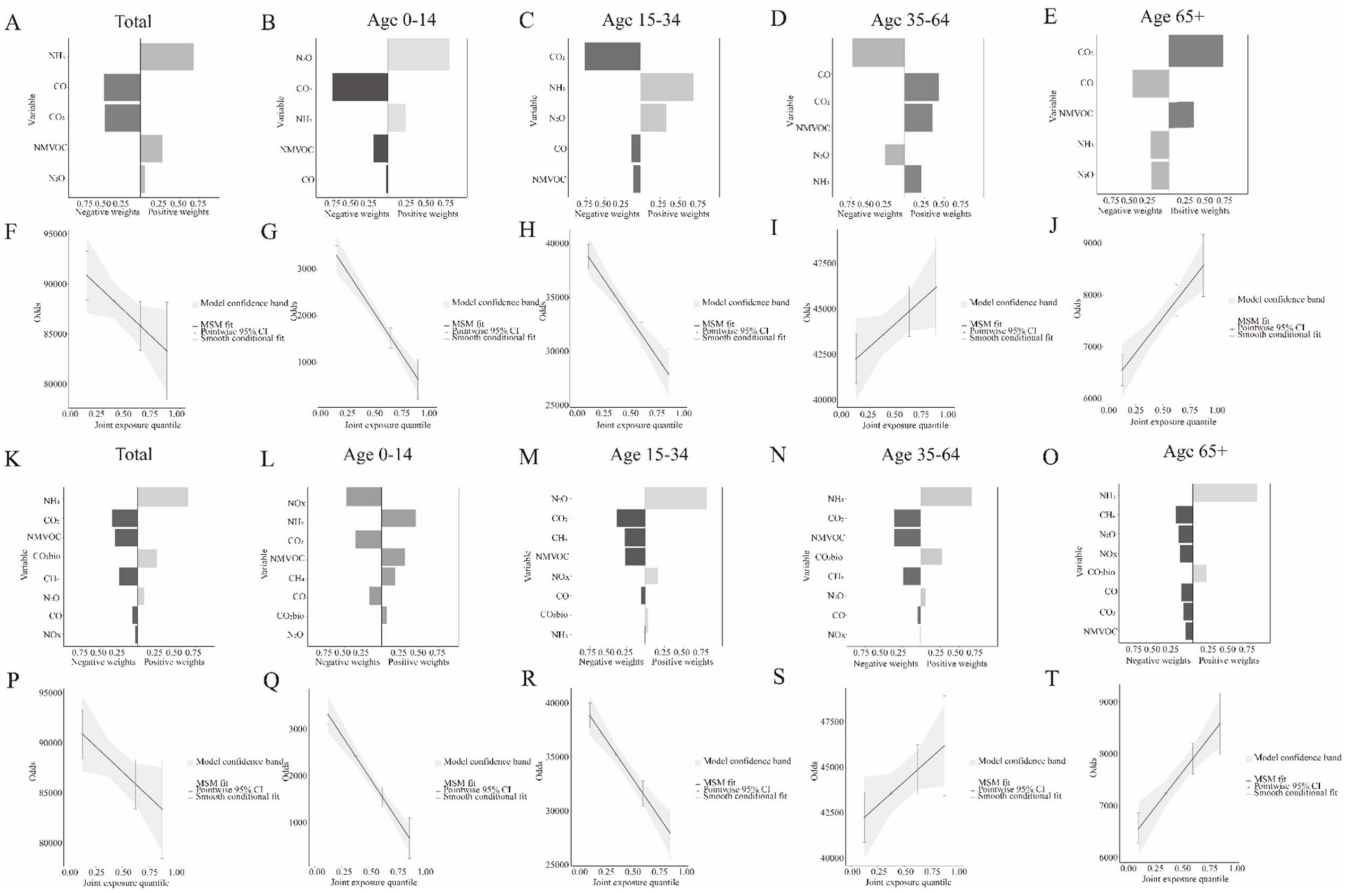
The index weights and joint effect (95 %CI) of the mixture of mixtures factors on HBV risk in cases of incidence and mortality for different age groups. The weights of mixtures on HBV incidence included **A** to **E**. The joint effect of mixtures on HBV incidence included **F** to **J**. The weights of mixtures on HBV mortality included **K** to **O**. The joint effect of mixtures on HBV mortality included **P** to **T**.

Figures 2A–E show the magnitude and direction of positive and negative contributions of greenhouse gases and air pollutants to HBV incidence in the overall population and across different age groups. Figures 2F–J show that the mixture of greenhouse gases and air pollutants exhibited a negative dose–response relationship with HBV incidence in the overall population and in younger patients (under 34 years of age), whereas a positive relationship was observed in older patients. Figures 2K–O show the magnitude and direction of positive and negative contributions of greenhouse gases and air pollutants to HBV-related deaths in the overall population and across different age groups. Figures 2P–T show that the mixture of greenhouse gases and air pollutants had a negative dose–response relationship with HBV mortality in the overall population and among younger patients (under 34 years of age), whereas a positive association was observed in older patients.

**Figure 3:**
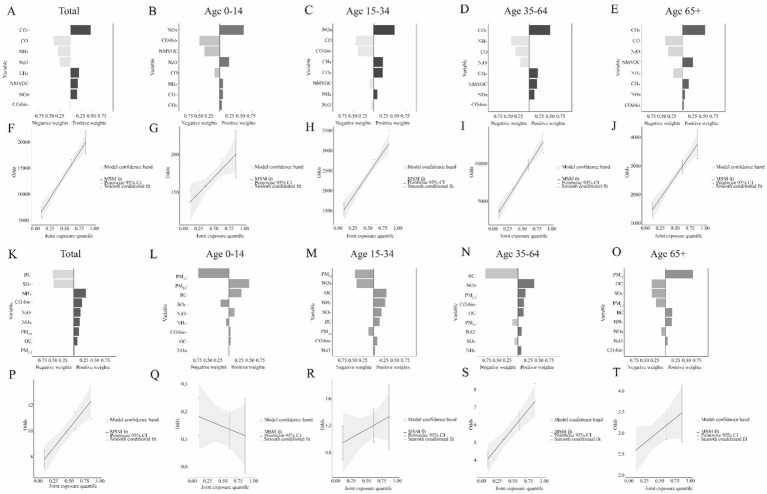
The index weights and joint effect (95 %CI) of the mixture of mixtures factors on HCV risk in cases of incidence and mortality for different age groups. The weights of mixtures on HCV incidence included **A** to **E**. The joint effect of mixtures on HCV incidence included **F** to **J**. The weights of mixtures on HCV mortality included **K** to **O**. The joint effect of mixtures on HCV mortality included **P** to **T**.

Figures 3A–E show the magnitude and direction of positive and negative contributions of greenhouse gases and air pollutants to HCV incidence in the overall population and across different age groups. Figures 3F–J show that a mixture of greenhouse gases and air pollutants had a positive dose–response relationship with HCV incidence across all age groups. Figures 3K–O show the magnitude and direction of positive and negative contributions of greenhouse gases and air pollutants to HCV-related deaths in the overall population and across different age groups. Figures 3P–T show that the mixture of greenhouse gases and air pollutants exhibited a negative dose–response relationship with HCV mortality in very young patients (under 14 years of age) and a positive dose–response relationship in the remaining age groups.

#### 3.2.2 Combined effects of multiple greenhouse gases and pollutants on hepatitis viruses: BKMR model

**Figure 4:**
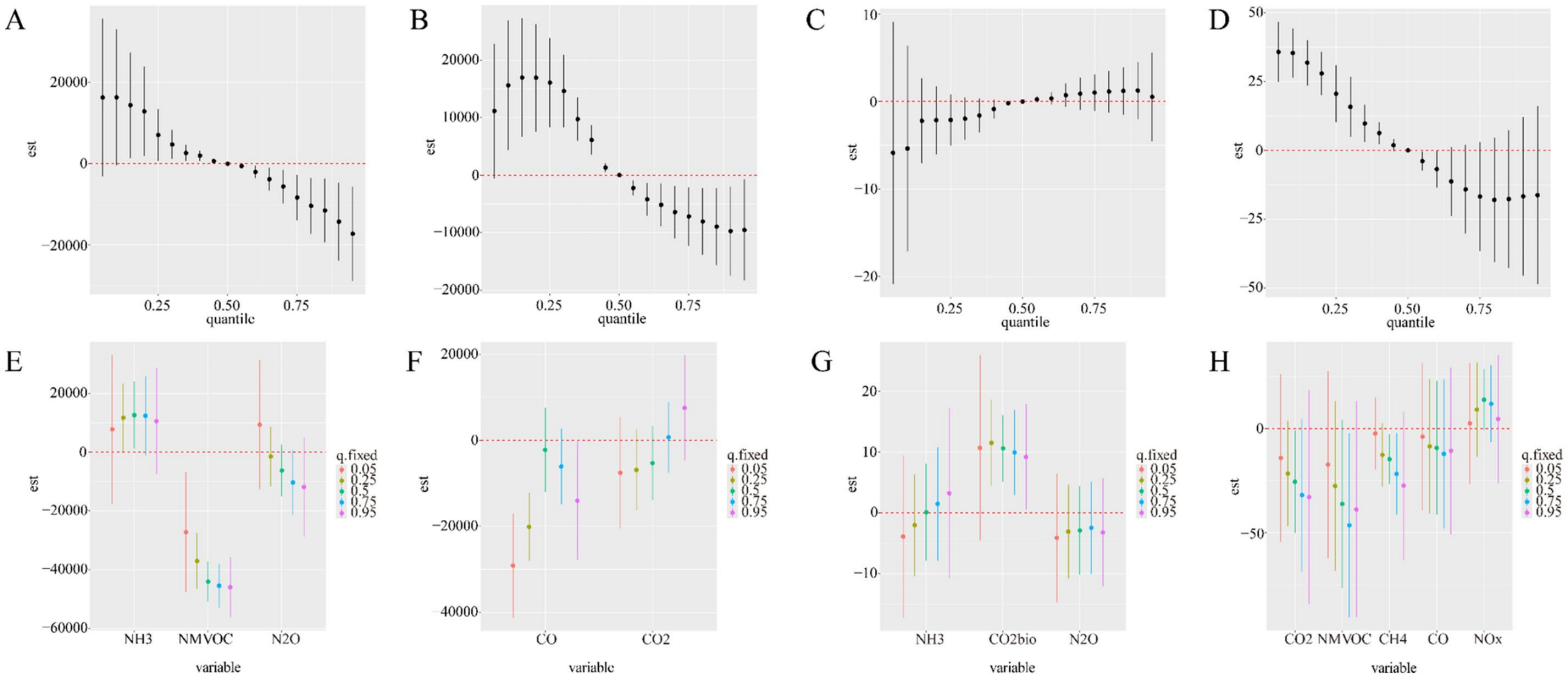
Associations between the mixtures of environmental factors and HBV incidence and mortality in the study population using the BKMR model. The model was adjusted for opposing factors using the Qgcomp model. **(A,B)** Cumulative effects of the mixtures on HBV incidence (estimates and 95% credible intervals). Mixtures are shown at specific percentiles (X-axis) compared to all exposures at the 50th percentile. **(C,D)** Cumulative effects of the mixtures on HBV mortality (estimates and 95% credible intervals). Mixtures are shown at specific percentiles (*X*-axis) compared to all exposures at the 50th percentile. **(E,F)** Single-exposure effects on HBV incidence (estimates and 95% credible intervals). **(G,H)** Single-exposure effects on HBV mortality (estimates and 95% credible intervals).

When the positive effector of HBV identified by Qgcomp (NH_3_, NMVOC, N_2_O) was considered as the main effect, the BKMR model output (Figure 4A) showed that HBV incidence decreased with increasing levels of GHG and air pollutant mixtures (16281.52 to −17227.94), with the strongest effect observed at low levels of the mixture (P15). As shown in Figure 4E, when considering single effects, NH_3_ (P25-P50) at low concentration levels exhibited a significant positive association with HBV morbidity, while NMVOC showed a significant negative association with morbidity across all concentration levels. When the negative effector of HBV (CO, CO_2_) identified by Qgcomp was considered as the main effect, the BKMR model output (Figure 4B) showed that the incidence of HBV decreased sharply with initial increases in the greenhouse gas and air pollutant mixture (range: 11165.35 to −9554.82). The strongest association was observed at low mixture levels (P15). As shown in Figure 4F, when considering single effects, CO (P5-P25) at low concentration levels exhibited a significant negative effect on HBV morbidity.

When the positive effectors of HBV-related deaths identified by Qgcomp (NH_3_, CO_2_bio, N_2_O) were considered as the main effects, the BKMR model output (Figure 4C) showed that HBV mortality first decreased and then increased with increasing levels of the greenhouse gas and air pollutant mixture. As shown in Figure 4G, CO_2_bio (P25-P95) showed a significant positive association with HBV mortality across nearly all concentration levels, with the association decreasing as concentration increased (maximum association (95%CI) = 11.54 (4.49, 18.60)). When the negative effectors of HBV-related deaths identified by Qgcomp (CO_2_, NMVOC, CH_4_, CO, NOx) were considered as the main effects, the BKMR model output (Figure 4D) showed that HBV mortality decreased with the increasing levels of the greenhouse gas and air pollutant mixture. The strongest associations were concentrated at low mixture levels (P5). As shown in Figure 4H, when considering single effects, CO_2_, NMVOC, and CH_4_ showed significant negative associations with HBV mortality at higher concentration levels (P25-P75).

**Figure 5:**
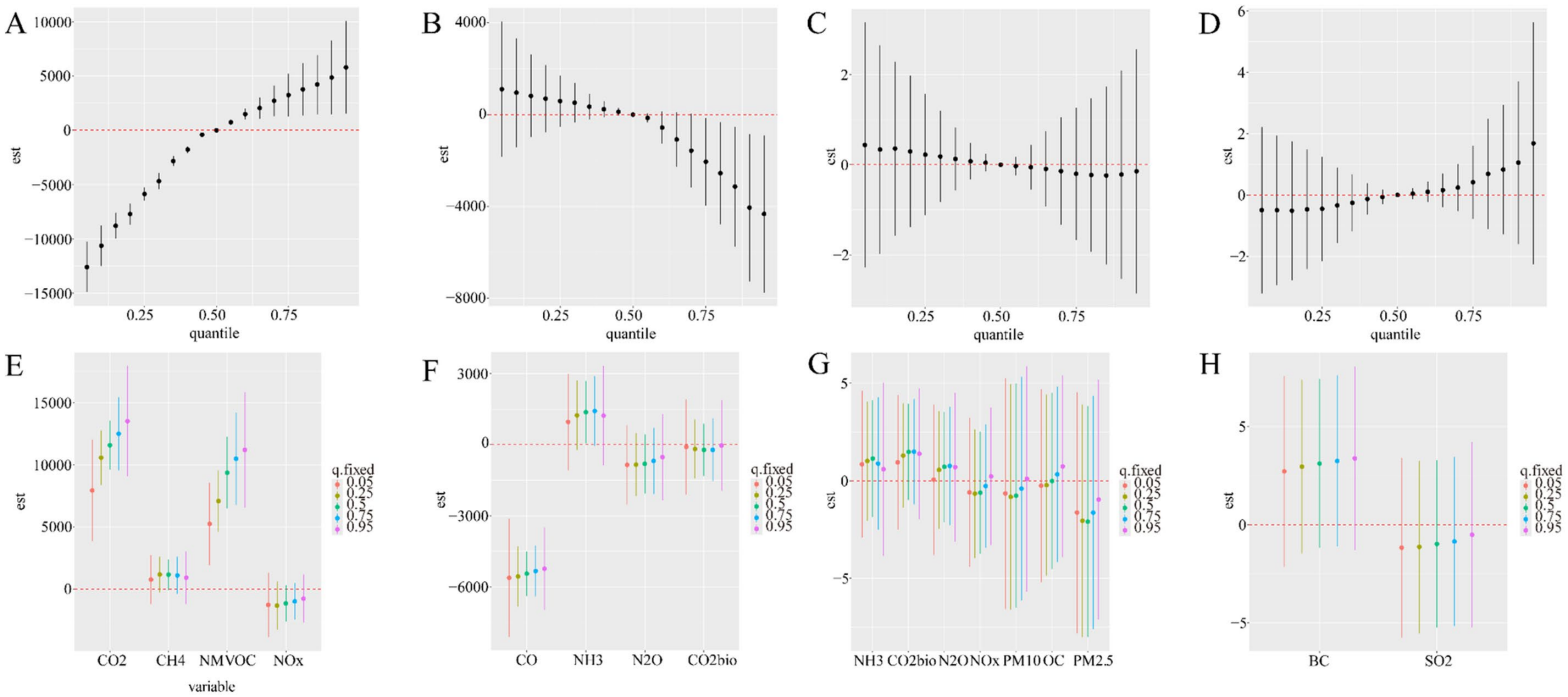
Associations between the mixtures of environmental factors and HCV incidence and mortality in the study population using the BKMR model. The model was adjusted for opposing factors using the Qgcomp model. **(A,B)** Cumulative effects of the mixtures on HCV incidence (estimates and 95% credible intervals). Mixtures are shown at specific percentiles (*X*-axis) compared to all exposures at the 50th percentile. **(C,D)** Cumulative effects of the mixtures on HCV mortality (estimates and 95% credible intervals). Mixtures are shown at specific percentiles (*X*-axis) compared to all exposures at the 50th percentile. **(E,F)** Single-exposure effects on HCV incidence (estimates and 95% credible intervals). **(G,H)** Single-exposure effects on HCV mortality (estimates and 95% credible intervals).

When the positive effectors identified by Qgcomp (CO_2_, CH_4_, NMVOC, and NOx) were considered as the main effects, the BKMR model output (Figure 5A) showed that HCV incidence increased with rising levels of the greenhouse gas and air pollutant mixture (from 746.35 to 5794.77), with the strongest associations observed at high mixture levels (P95). As shown in Figure 5E, when considering single effects, CO_2_ and NMVOC exhibited significant positive associations with HCV morbidity across all concentration levels, with the strength of the association increasing as concentration increased. When the negative effectors of HCV incidence identified by Qgcomp (CO, NH_3_, N_2_O, and CO_2_bio) were considered as the main effects, the BKMR model output (Figure 5B) showed that HCV incidence decreased with increasing levels of the greenhouse gas and air pollutant mixture. As shown in Figure 5F, when considering single effects, CO exhibited a significant negative association with HCV morbidity across all concentration levels, while NH_3_ showed a significant positive association with morbidity at moderate concentrations.

When the positive effectors (NH_3_, CO_2_bio, N_2_O, NOx, PM_10_, OC, PM_2.5_) and negative effectors (BC, SO_2_) of HCV mortality identified by Qgcomp were considered as the main effects, the BKMR model output (Figures 5C,D) showed that the effect on HCV mortality did not change with the levels of the greenhouse gas and air pollutant mixture. When considering single effects, as shown in Figures 5G,H, there were no significant associations between greenhouse gases or air pollutants and HCV mortality at any concentration level.

According to the Qgcomp and BKMR models, the relative contributions of different greenhouse gases and air pollutants to the incidence and mortality of HBV and HCV are shown in Table 4. The main pollution factors affecting the incidence of HBV were NH_3_ (PIP_Qgcomp_ = 0.6731) and CO (PIP_BKMR_ = 0.9966), while the main pollution factors affecting HBV mortality were NH_3_ (PIP_Qgcomp_ = 0.6597) and NMVOC (PIP_BKMR_ = 0.9581). The main pollution factor affecting the incidence of HCV was CO_2,_ while the main pollution factor affecting HCV mortality was BC.

**Table 4:** Posterior inclusion probabilities (PIPs) for pollution mixtures in different models for HBV and HCV, using the quantile-based g-computation (Qgcomp) and Bayesian kernel machine regression (BKMR) models.

Series	Direction	Pollutants	PIPs	
			Qgcomp	BKMR
HBV cases	Positive	NH_3_	0.6731	0.7288
		NMVOC	0.275	0.9914
		N_2_O	0.0518	0.647
	Negative	CO	0.505	0.9966
		CO_2_	0.495	1
HBV deaths	Positive	NH_3_	0.6597	0.1959
		CO_2_bio	0.252	0.7739
		N_2_O	0.0883	0.0717
	Negative	CO_2_	0.3456	0.8311
		NMVOC	0.3029	0.9581
		CH_4_	0.2505	0.8843
		CO	0.0691	0.8877
		NOx	0.0319	0.9021
HCV cases	Positive	CO_2_	0.475	1
		CH_4_	0.198	0.2706
		NMVOC	0.17	0.8284
		NOx	0.157	0.1654
	Negative	CO	0.4108	0.9992
		NH_3_	0.3144	0.1215
		N_2_O	0.2595	0.0675
		CO_2_bio	0.0154	0.0337
HCV deaths	Positive	NH_3_	0.2828	0.3549
		CO_2_bio	0.1917	0.3078
		N_2_O	0.1562	0.3203
		NOx	0.1394	0.3024
		PM_10_	0.1261	0.3874
		OC	0.089	0.2963
		PM_2.5_	0.0148	0.4538
	Negative	BC	0.51	0.7128
		SO_2_	0.49	0.4404

### 3.3 Multiple prediction

It can be seen from the different prediction effects of GBRT on HBV and HCV morbidity and mortality in Table 5 and Figure S2. For greenhouse gases and air pollutants as predictors, the prediction effect of HCV morbidity was the best (R^2^Training = 0.950, R^2^Test = 0.942). The accuracy of the prediction line is very similar between the training set and the test set, and the fitting values basically fall into the prediction line. The second-best predictor was the prediction of HBV death. However, in predicting HCV death, R^2^∈ [0.027, 0.287]. Figure S2 corresponds to Table 5 in this respect.

**Table 5:** Comparison of internal validation for HBC and HCV prediction using the gradient boosting regression tree (GBRT).

Model series	Training set			Test set		
	RMSE	R^2^	MAE	RMSE	R^2^	MAE
HBV cases	7144.675	0.621	5746.536	9342.574	0.464	7083.872
HBV deaths	5.681	0.918	4.516	9.759	0.772	7.963
HCV cases	1108.988	0.950	856.374	1252.090	0.942	927.450
HCV deaths	3.801	0.287	3.130	3.766	0.027	3.024

## 4 Discussion

In China, there has been an overall downward trend in the prevalence of viral hepatitis, particularly HBV. This decline may be due to the reuse of contaminated needles and mixed blood components in the past century, which contributed to the spread of blood-borne hepatitis (16). In 2020, American virologists Harvey J. Alter and Charles M. Rice and British virologist Michael Houghton won the Nobel Prize in Physiology or Medicine for their discovery of HCV, which accelerated global efforts in the development and implementation of HCV vaccines. However, only a small proportion of people in our country are aware of HCV, which is much lower than the awareness of HAV and HBV. Such neglect also makes HCV in this study, of course, also presents an increasing trend year by year. This study found that HBV and HCV morbidity and mortality were highest among individuals aged 35–64 years, consistent with the results of a study conducted in Shenyang, China (17). Currently, HBV and HCV are primarily transmitted through blood. Individuals around 35 years old who are sexually active, as well as young laborers in households, represent high-risk groups. Due to insufficient attention to health and preventive measures, illness and death from these infections constitute the main burden of hepatitis in China (18). Regarding the seasonal characteristics of viral hepatitis, this study found that its prevalence in China is highest during spring and summer, likely influenced by seasonal environmental conditions. During spring and summer, the gradual rise in temperature and increase in ultraviolet radiation intensity create conditions that promote bacterial growth in food and may lead to reduced human immunity. These factors collectively contribute to the exacerbation of hepatitis incidence and severity. At the same time, the long days and short nights can disrupt people’s daily routines, and unhealthy eating habits increase the burden on the liver, contributing to a higher risk of hepatitis and related mortality. HBV incidence is primarily concentrated among young people, and it is significantly higher than in older adults. This may be due to the low vaccination coverage among the older population, coupled with a high rate of immune consolidation after natural infection (high anti-HBc positivity rate), which results in a lower risk of reinfection. In contrast, although young people have high vaccination coverage, they remain the primary group for new infections (17).

Through Qgcomp analysis, we found that the risk of developing or dying from HBV increases with higher levels of greenhouse gases and air pollutants, particularly among young adults over 35 years and older adults over 65 years. Studies have shown that air pollution, especially pollutants such as fine particulate matter (PM_2.5_) and nitrogen dioxide (NO_2_), can damage the body’s immune system. Air pollutants can enter the body through the respiratory tract, trigger inflammatory responses, weaken immune function, and make older adults more susceptible to infections such as HBV, while reducing viral clearance efficiency (19), which is consistent with our findings. Our study confirms that environmental factors still have a positive association with HCV morbidity and mortality across different age groups. These findings highlight that, in addressing the challenge of HCV, China still requires the engagement of the entire population and coordinated preventive measures.

Through BKMR analysis, this study found that CO_2_ and CH_4_ were the primary greenhouse gases associated with increased risk of viral hepatitis and mortality, mainly because one of the main consequences of rising CO_2_ is climate change, particularly global temperature increase. These changes have a certain effect on the human immune system (20). Extreme weather events caused by climate change, such as high temperatures and humidity changes, can affect people’s health, especially older adults and people with weakened immune systems. When immunity is compromised, the body’s ability to resist viral infections is reduced. Infections such as HBV are more likely to develop into chronic or severe disease when the immune system is suppressed, exacerbating liver damage and increasing the risk of mortality. At typical atmospheric concentrations, environmental greenhouse gases may also directly affect the outcome of viral hepatitis. This study incorporates greenhouse gases as a comprehensive exposure indicator by integrating meteorological factors and air pollutants, allowing for a more comprehensive exploration of the environmental factors’ impact on the risk of hepatitis. A study in the United States showed that greenhouse gas emissions influence respiratory viral infections (such as influenza and RSV) through multiple pathways. The study also highlighted the dual integration attributes of greenhouse gases, both driving meteorological changes and being co-emitted with other pollutants (21). A study in Taiwan found some variation in the association between O_3_ levels and liver fibrosis in late-stage HBV patients (22), which may be because long-term exposure to air pollutants may increase oxidative stress, promote chronic inflammatory responses, and further exacerbate liver damage. In patients with HBV, oxidative stress may accelerate the liver fibrosis process and even lead to cirrhosis. Therefore, although CH_4_ identified in this study does not directly cause hepatitis, it can react with oxygen in the atmosphere to produce pollutants such as O_3_. Ozone and other air pollutants (such as PM_2.5_) may have a negative impact on liver health.

This study also found that NMVOC and NH_3_ were the main air pollutants associated with an increased risk of viral hepatitis and mortality. The accumulation of ammonia can interfere with the normal metabolic process of cells, resulting in cell damage, apoptosis, and even necrosis, thereby further aggravating the decline in liver function. It also stimulates immune cells (such as macrophages and T cells) to produce an inflammatory response and promote the release of inflammatory factors (such as cytokines and chemokines) (23). In patients with hepatitis, this excessive inflammatory response may aggravate liver damage and promote the progression of liver fibrosis. Chronic inflammation is one of the important factors in the progression of liver disease, especially in chronic HBV and HCV. Among NMVOC emissions, 16.7% were caused by 15 carcinogenic compounds. In particular, methanol, formaldehyde, 2-propanol, and ethanol accounted for 55.5, 16.6, 11.7, and 8.31% of NMVOC emissions, respectively (24). At present, there are few studies on the effect of NMVOCs on population health, largely because the exposed population mainly consists of specific occupational groups, and the level of cooperation and study completion among this population is very low. Many studies have shown that follow-up in this population is poor, and the course of most related diseases is long, which makes it very difficult to carry out research. This study identified its external environmental impact and hopes to further analyze the internal mechanism in the future.

Although this study introduced new greenhouse gases into the environmental factors associated with viral hepatitis and included previously neglected air pollutants, it lacked certain social and demographic characteristics when adjusting for confounders. Although the study has issues with factor collinearity, a mixture model was applied to select positive factors for subsequent analysis to examine the overall impact of environmental factors on hepatitis, which can effectively reduce the influence of highly correlated variables. However, multiple studies have shown that selecting factors based on multicollinearity can effectively improve the accuracy of research findings (25). In the preliminary phase of the study, the variance inflation factor (VIF) analysis showed that some variables had VIF values greater than 5 (the critical threshold), indicating the presence of multicollinearity issues in the model. It is hoped that future adjustments will incorporate sufficient confounding factors to mitigate this collinearity issue. Although the Qgcomp model initially used mixed weights that could not reflect the true causal importance, a phased analysis was conducted, followed by the use of a Bayesian model. The Bayesian mixture model, by incorporating prior information, can constrain the coefficient estimates of collinear variables, reducing unreasonable statistical weight distribution and better aligning with the true causal effects. In addition, our study only identified associations and characteristics of viral hepatitis in the external environment and lacked internal exposure measurements, so the results based on existing data may be overestimated. Future hematological studies of exposed individuals are needed to address this limitation.

This study was conducted at the ecological level and did not adjust for key sociodemographic and healthcare-related factors, which may introduce a certain risk of confounding bias. The observed associations may reflect broader socioeconomic or healthcare system differences rather than the direct effects of environmental factors. Moreover, the environmental variables represented emission levels rather than actual individual exposure levels, which may have led to exposure misclassification bias. Future research should integrate environmental monitoring data, census data, healthcare resource statistics, and public health surveillance data to comprehensively adjust for key sociodemographic factors (such as age, sex, socioeconomic status, and population density) and healthcare-related factors (such as vaccination coverage, medical resource accessibility, and diagnostic capacity). This would help eliminate residual confounding and more accurately estimate the independent effects of environmental factors. In addition, future studies should conduct individual-level cohort or case–control studies to collect detailed data on personal environmental exposure levels (e.g., individual PM2.5 and O₃ exposure monitoring), sociodemographic characteristics, healthcare utilization, and viral infection outcomes. Such designs would enable the validation of the association between environmental factors and infection outcomes at the individual level, thereby avoiding ecological fallacy and enhancing the reliability of causal inference.

Although this study incorporated greenhouse gases, which simultaneously capture dual attributes (meteorological and pollution-related characteristics), the analytical framework relied on climate-mediated mechanisms. Therefore, future research should incorporate specific meteorological variables or conduct mediation analyses to further support this proposed pathway. Furthermore, the study did not explicitly address the temporal lag between exposure and outcome. Given the slow progression of viral hepatitis, modeling exposure and outcome within the same time window may not reflect biologically plausible associations. Future analyses should therefore account for potential lag effects.

## 5 Conclusion

We found that most greenhouse gases and air pollutants are associated with increased HBV and HCV morbidity and mortality in China. The relationship between environmental factors and hepatitis varied substantially across different age groups and hepatitis types. These findings may have important implications for the development of effective public health interventions and integrated early warning systems targeting air pollution and viral hepatitis.

## Data Availability

Publicly available datasets were analyzed in this study. The data that support the findings of this study are available on request from the National Public Health Data Centre of China (https://www.phsciencedata.cn/) and the Emissions Database for Global Atmospheric Research (EDGAR) (https://edgar.jrc.ec.europa.eu/).
