# Correction: Impact of greenhouse gases and air pollutants on the incidence and mortality of HBV and HCV in China

**DOI:** 10.3389/fpubh.2026.1855725

**Published:** 2026-04-29

**Authors:** Shengfei Pei, Fan Shi, Qinglin Li, Bo Zhu, Shumei Huang, Xin Zhang

**Affiliations:** 1 Changqing University Science Park, Shandong Women's University, Jinan, China; 2 School of Basic Medical Sciences, Health Science Center, North China University of Science and Technology, Tangshan, China; 3 Department of Public Health, Tianjin Union Medical Center (First Affiliated Hospital of Nankai University), Tianjin, China; 4 The First Affiliated Hospital of Shandong First Medical University, Jinan, China; 5 Hebei Key Laboratory of Immune Mechanism of Major Infectious Diseases and New Technology of Diagnosis and Treatment, The Fifth Hospital of Shijiazhuang, Shijiazhuang, China; 6 Department of Tuberculosis, The Fifth Hospital of Shijiazhuang, Shijiazhuang, China

**Keywords:** Bayesian kernel machine regression, gradient boosted regression trees, greenhouse gases, mixtures analysis, viral hepatitis

The funder “Medical Science Research Project of Hebei Province (20260942)” was erroneously omitted. The correct **Funding** statement reads:

“The author(s) declared that financial support was received for this work and/or its publication. This work was funded by Medical Science Research Project of Hebei Province (20260942).”

The original version of this article has been updated.

